# Overexpression of a *Triticum aestivum* Calreticulin gene (*TaCRT1*) Improves Salinity Tolerance in Tobacco

**DOI:** 10.1371/journal.pone.0140591

**Published:** 2015-10-15

**Authors:** Yang Xiang, Yun Hai Lu, Min Song, Yun Wang, Wenqi Xu, Lintao Wu, Hancheng Wang, Zhengqiang Ma

**Affiliations:** 1 Guizhou Rapeseed Institute, Guizhou Academy of Agricultural Sciences, Guiyang, China; 2 College of Crop Science, Fujian Agriculture and Forestry University, Fuzhou, China; 3 Crop Genomics and Bioinformatics Center and National Key Laboratory of Crop Genetics and Germplasm Enhancement, Nanjing Agricultural University, Nanjing, China; 4 Qufu Normal University, College of Life Sciences, Qufu, China; Institute of Genetics and Developmental Biology, Chinese Academy of Sciences, CHINA

## Abstract

Calreticulin (CRT) is a highly conserved and abundant multifunctional protein that is encoded by a small gene family and is often associated with abiotic/biotic stress responses in plants. However, the roles played by this protein in salt stress responses in wheat (*Triticum aestivum*) remain obscure. In this study, three *TaCRT* genes were identified in wheat and named *TaCRT1*, *TaCRT*2 and *TaCRT3-1* based on their sequence characteristics and their high homology to other known *CRT* genes. Quantitative real-time PCR expression data revealed that these three genes exhibit different expression patterns in different tissues and are strongly induced under salt stress in wheat. The calcium-binding properties of the purified recombinant *TaCRT1* protein were determined using a PIPES/Arsenazo III analysis. *TaCRT1* gene overexpression in *Nicotiana tabacum* decreased salt stress damage in transgenic tobacco plants. Physiological measurements indicated that transgenic tobacco plants showed higher activities of superoxide dismutase (SOD), peroxidase (POD) and catalase (CAT) than non-transgenic tobacco under normal growth conditions. Interestingly, overexpression of the entire *TaCRT1* gene or of partial *TaCRT1* segments resulted in significantly higher tolerance to salt stress in transgenic plants compared with their WT counterparts, thus revealing the essential role of the C-domain of *TaCRT1* in countering salt stress in plants.

## Introduction

The Calreticulin protein (CRT) was first identified as a high affinity Ca^2+^-binding protein from the endoplasmic reticulum (ER) of rabbit skeletal muscle in 1974 [1]. In 1989, rabbit [2] and mouse [3] forms of this protein were cloned. Since then, CRT genes have been isolated from various other species including humans [4], sheep [5], nematodes [6], fruit flies [7], spinach [8], barley [9], tobacco (*Nicotiana benthamiana*) [10], corn [11–13], Chinese cabbage (*Brassica pekinensis*) [14], *Arabidopsis thaliana* [15], castor beans [16], rice [17], wheat [18,19], sea bass [20] and *Anopheles stephensi* [21]. CRT genes are present in all studied multicellular eukaryotes and are highly conserved [22]. However, these genes have not been found in yeast or prokaryotes, suggesting that *CRT* genes evolved shortly before plants and animals diverged during the evolutionary process.

A typical CRT protein comprises three distinct subdomains: a conserved globular N-domain including a cleavable signal sequence, which directs the protein to the ER, a P-domain, which exhibits high affinity, low capacity Ca^2+^-binding ability and a C-terminal domain, which includes a (K/H)DEL ER retrieval signal [22,23] and exhibits low affinity, high capacity Ca^2+^-binding ability. Inside the cells, CRTs are mainly present in the ER [24] but also in the nucleus [25], the nuclear envelope [12], the cytosol [26], the spindle apparatus of dividing cells [10], the cell surface [27], mitochondria [28] and plasmodesmata [29,30], indicating that CRTs are important for several cellular functions.

Extensive studies of mammalian CRTs have defined more than 40 physiological functions inside and outside the ER [22,23,31,32], including intracellular Ca^2+^ storage, the regulation of ER Ca^2+^ homeostasis [33–35], involvement in Ca^2+^-dependent signal pathways [36–39], molecular chaperone activity in the ER [40–45], control of cell adhesion [24,46], angiogenesis [47], functions related to the immune system and apoptosis [48] as well as roles in pathogenesis [49].

Despite the elucidation of CRT functions in animal cells, the role of plant CRTs is less clear [50]; however, plant CRTs have been shown to bind to calcium in the same way as their animal homologs [9,16,17,51]. In addition, plant CRTs exhibit calcium-storing functions in the ER of plant cells [35,52]. Recently, it was demonstrated that plant CRTs are able to modulate intracellular Ca^2+^ homeostasis and to function in the ER quality control (ERQC) of N-glycosylated proteins [53–55]. Moreover, plant CRTs exhibit unique features, as demonstrated by newly obtained data. For example, plant CRTs might play a role in the response to some phytohormone stimuli [10,17,56], pollen-pistil interactions [57], the regulation of root and shoot regeneration processes [17] and plant immunity [43,54]. In addition, plant CRTs respond to a variety of stress-mediated stimuli, e.g., cold and gravi-stimulation [58–60], indicating that plant CRTs play multiple roles in plant development and stress responses.

In the ‘Hanxuan 10’ variety of wheat, Jia et al. [18] isolated a full-length cDNA that encodes a CRT3 isoform of the calreticulin protein family (named *TaCRT*). The subcellular location of this isoform was determined to be the cytoplasm and the nucleus by transiently expressing GFP fused with TaCRT in onion epidermal cells. The CRT3 isoform transcript is up-regulated in wheat seedlings by PEG-induced drought stress. Moreover, *TaCRT* overexpression resulted in an enhanced drought resistance to several water deficit conditions in tobacco.

In this study, three new full-length cDNAs encoding wheat CRT1, CRT2 and CRT3 isoforms were isolated and named *TaCRT1*, *TaCRT2* and *TaCRT3-1*, respectively. These isoforms were obtained from spike tissues of the ‘Wangshuibai’ hexaploid wheat cultivar using reverse transcription PCR (RT-PCR). The expression patterns of these isoforms were determined in various organs and under salinity stress conditions in wheat. The calcium-binding properties of purified recombinant TaCRT1 protein were determined through PIPES/Arsenazo III analysis. Interestingly, transgenic tobacco plants overexpressing the entire *TaCRT1* or partial *TaCRT1* segments exhibited significantly greater tolerance to salt stress than their WT counterparts, revealing that the C-domain of *TaCRT1* is essential for the response to salt stress in plants.

## Materials and Methods

### Plant Materials and Growth Conditions

The plants used in this study included the hexaploid wheat cv. Wangshuibai and *Nicotiana tabacum* cv. SamSun. Wangshuibai plants were planted in a field at the experimental station of Nanjing Agricultural University, Nanjing, China, unless otherwise indicated. Tobacco plants were planted in a controlled environment chamber (150 μmol photons m^-2^ s^-1^, 16 h light/8 h dark per day at 25/16°C). Wheat roots, stems, leaves, glumes, inner bracts, outer bracts, pistils, stamens, rachises and awn tissues were collected from field-planted wheat 15 d after anthesis. Six-day-old seedlings were transferred to Petri dishes containing 250 mM NaCl (Nanjing Chemical Reagent) to inflict osmotic stress conditions. The seedlings were grown at room temperature under 16 h of light daily. The root tissues were harvested at 0.5, 3, 6, 9, 12, and 24 h after the transfer to Petri dishes.

### Isolation and Sequence Analysis of *TaCRT*s

The plant CRT proteins were queried against a wheat dbEST (1,071,054 ESTs, the 177^th^ release of GenBank, 2010) using the function tBlastn. Using the criteria of >95% identity and E<e-100, EST hits for wheat *CRT* genes were retrieved and used in contig assembly with the parameter settings of > 40 bp overlap and > 95% identity after removing possible vector sequence contamination. Genomic DNA was extracted according to the procedure described by Ma et al. [61]. Total RNA was extracted from the wheat samples using Trizol reagent (Invitrogene, USA) following the manufacturer’s protocol and quantified using a spectrometer (Ultrospec 2100 pro, Amersham Pharmacia, England). First strand cDNA was synthesized from 3 μg of total RNA using Moloney murine leukemia virus reverse transcriptase (Promega, USA) and oligo (dT15) primers according to the manufacturer’s instructions. RT-PCR was performed in a 25-μl mixture containing approximately 5 ng of template, 5 pmol of each primer, 5 nmol of each dNTP, 37.3 nmol MgCl_2_, and 0.5 U Taq DNA polymerase (TaKaRa, Kyoto, Japan). The PCR conditions used were as follows: 94°C for 3 min followed by 30 cycles of 94°C for 20 s, 58°C for 30 s, and 72°C for 1.5 min. The primers used for the RT-PCR are listed in S1 Table. The sequencing was performed by Invitrogen Corporation, Shanghai, China, and the nucleotide sequences were searched for in the NCBI databases.

Open reading frame (ORF) identification, protein translation prediction, molecular mass (MW) calculation and sequence alignment were conducted using Macvector 10.0 software (Accelrys, Oxford, USA). The signal peptide was predicted using WoLF PSORT (http://psort.nibb.ac.jp/), and the conserved domain was predicted using SMART (http://smart.embl-heidelberg.de). A phylogenetic analysis was conducted using the neighbor-joining algorithm included in MEGA4.0 software [62]. The robustness of the phylogenetic tree topology was assessed based on bootstrap values. *TaCRT* sequences were individually mapped over wheat genome at International Wheat Genome Sequencing Consortia (IWGSC, http://wheat-urgi.versailles.inra.fr/Seq-Repository) [63, 64].

### Quantitative Real-time PCR Analysis

Quantitative real-time PCR (qRT-PCR) was performed using a SYBR-Green PCR Mastermix (ToYoBo, Osaka, Japan) and a Bio-Rad iCYCLER iQ5 (Bio-Rad, USA); the 25-μL reactions contained approximately 5 ng of cDNA template, 12.5 μL of SYBR-Green PCR Mastermix (ToYoBo, Osaka, Japan) and 10 pmol of each primer. Each sample was analyzed in triplicate. Data were normalized using the α-*Tubulin* gene from wheat as the reference [65]. Relative expression was estimated using the 2^−ΔΔCt^ method [66]. Three biologically independent experiments were performed. The primer sequences used are listed in S2 Table.

### Expression and Purification of Recombinant Proteins and Calcium-binding Analysis

An *Escherichia coli* expression plasmid was constructed using full-length cDNA from *TaCRT1*, which was amplified using the following primers: forward 5'-AAGGATCCATGGCGATCCGCCGTG-3', reverse 5'CCGTCGACCATCCATTTAGAGCTCATCGTG-3' (*BamH*I and *Sal*I sites are underlined). The PCR products were digested with *BamH*I and *Sal*I and inserted into a pET32a IPTG-inducible expression vector (Qiagen Inc., Chatsworth, CA). The construct was expressed in *E*. *coli* host strain BL21 (DE3). The bacteria were cultured at 37°C until OD_600_ = 0.6, at which point IPTG (Merck Chemicals, Shanghai, China) was added to a final concentration of 1 mM; the culture was then further cultured at 37°C for an additional 3 h. Induced *E*. *coli* cells were pelleted by centrifugation at 12,000 g for 5 min and resuspended in extraction buffer (50 mM sodium phosphate buffer, pH 8.0, 3 M NaCl and 1 mM PMSF). The recombinant protein was purified by capturing its 6x histidine tag on a Ni-NTA column (nickel nitrilotriacetic acid; Qiagen Inc.) under denaturing conditions. The protein was purified according to the protocol provided by the manufacturer. The calcium-binding capacity of the purified recombinant TaCRT1 protein was determined using PIPES/Arsenazo III analysis [67].

### Construction of *TaCRT1* Plant Expression Vectors and Plant Transformation

To generate cauliflower mosaic virus (CaMV) 35S-driven constructs, the ORF and segments of *TaCRT* were amplified using *pfu* DNA polymerase (Dingguo Biotech, China) and the primers listed in S3 Table. The amplified *TaCRT1* included the ER import signal sequence (the SS region), the P-domain, the C-domain and the ER-retention signal HDEL. Restriction-digested PCR products were inserted into a modified pBI121 expression vector, which was kindly provided by Dr. Deyue Yu of Nanjing Agricultural University. The recombinant vector was then transformed into *Agrobacterium tumefaciens* strain LBA4404 by electroporation. The tobacco plants were transformed by the leaf disc method [68] using *Agrobacterium tumefaciens* strain LBA4404. Positive transgenic plants overexpressing *TaCRT1* were first screened on agar solidified with Murashige and Skoog salts [69] and 1% sucrose (MS medium) containing 150 mg/L of kanamycin, and then by PCR (S1A Fig) with the primers presented in S3 Table. The expression of *TaCRT1* or *TaCRT1* segments in T_2_ transgenic lines was detected by RT-PCR (S1B Fig) with the same set of primers presented in S3 Table. Homozygous T_2_ transgenic plants were identified by analyzing the segregation of T_3_ seeds germination on MS medium containing 150 mg/L of kanamycin.

### Salt Stress Treatments of Tobacco Plants

Seeds from homozygous T_3_ transgenic and non-transgenic tobacco plants were allowed to germinate in MS medium in the culturing room. Two-week-old seedlings were transferred into pots filled with a mixture of soil and vermiculite and given 16 h light/8 h dark per day at 25/16°C. Seedlings at the four-leaf stage were irrigated with saline water containing 150 mM or 250 mM NaCl every 2 d in the greenhouse for 20d to evaluate their salt tolerance. We use a less severe stress of 150 mM NaCl (according to Deng et al. [70]) for better evaluate the differences in root development and a more severe stress of 250 mM NaCl (according to Sheveleva et al. [71]) for better appreciate the differences in seedling development between transgenic and WT plants in response to salt stress. The length of the root and the weight of fresh roots were measured after the 20^th^ day of the NaCl treatment.

### Germination Assay

Seeds from T_3_ transgenic and non-transgenic tobacco plants were sown on filter paper in Petri dishes soaked with distilled water containing 150 mM NaCl. One hundred and fifty seeds were sown on three plates for each line. After stratification at 4°C for 2 d, the plates were moved to a chamber at room temperature and exposed to 15 h of light per day. Germination (emergence of the primary root) was scored daily until 6 days after sowing.

### Leaf Disc Assays and Measurement of Chlorophyll Contents

The leaf discs of 1 cm diameter were cut from the healthy, fully expanded leaves of transgenic and WT plants grown under unstressed condition and floated on 400 mM NaCl for 4 days according to Negi et al. [72]. For measurement of chlorophyll contents, ten leaf discs were thoroughly homogenized in 95% ethanol (v/v) and centrifuged at 3,000 ×g for 2–3 min. The O.D. of each supernatant was recorded at 663 and 645 nm using a spectrophotometer (PGENERAL UV-1800S, Beijing, China), and the chlorophyll content was calculated per gram of fresh tissue according to Arnon [73].

### Antioxidant Enzyme Activities

The activities of antioxidant enzymes such as peroxidase superoxide dismutase (SOD, EC 1.15.1.1), (POD, EC 1.11.1.7) and catalase (CAT, EC 1.11.1.6), were measured in the roots from potted seedlings at the four-leaf stage of WT and transgenic tobacco under normal growth conditions. The activities of SOD and CAT were measured as described in Beyer and Fridovich [74] and Corbisier et al. [75], respectively. POD activity was measured spectrophotometrically by monitoring the increase in absorbance at 460 nm following the method of Polle et al. [76].

### Statistical Analysis

The data sets were compared using the pairwise *t* test module included in Microsoft Office Excel 2008.

## Results

### Isolation and Characterization of *CRT* Genes from Wheat

**Fig 1:**
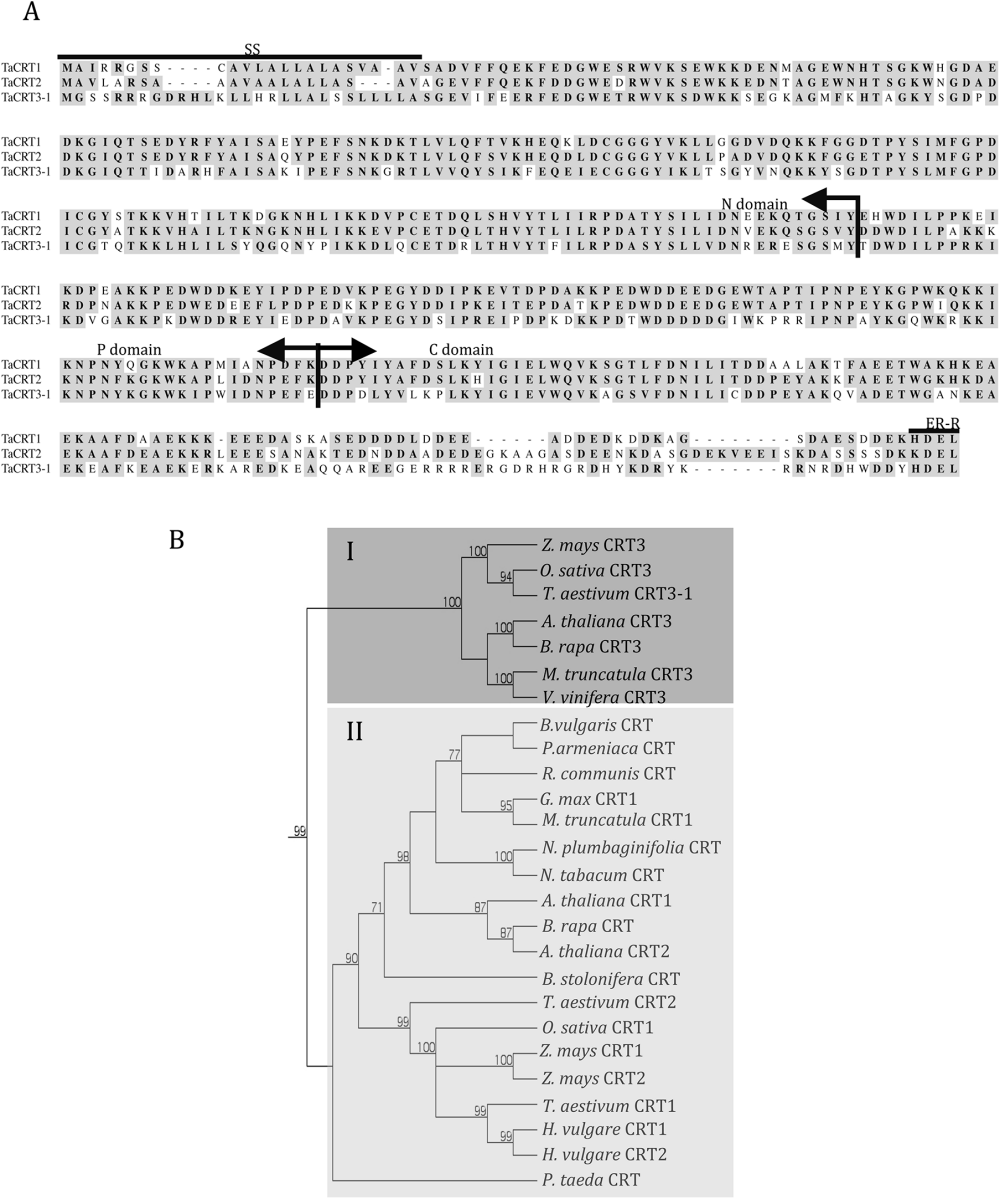
Primary structure comparison among wheat CRT proteins (A) and phylogenetic analysis of plant CRT proteins (B). Dark shading indicates conserved residues. SS, the ER import signal sequence; ER-R, the ER-retention signal HDEL. The protein sequences used to construct the phylogenetic tree are presented in S4 Table.

Publicly accessible wheat expressed sequence tag (EST) databases (http://www.ncbi.nlm.nih.gov/) were searched using the tBLASTn protocol with twenty-three plant CRT proteins (S4 Table) as queries. Three significant contigs with full-length ORFs (FL-ORFs) were obtained. Based on the sequence information, three pairs of gene-specific primers were designed. Three cDNA fragments (1473, 1473 and 1467 bp) covering FL-ORFs of corresponding *CRT* genes were obtained by PCR amplification from spike tissues of the Wangshuibai hexaploid wheat cultivar and were designated *TaCRT1* (Accession number: AY836753), *TaCRT2* and *TaCRT3-1*, respectively, according to their sequence similarity to the known *CRT* genes. BLAST searches against the wheat genome at IWGSC revealed that *TaCRT1* was located on chromosome arm 2DL (with two additional homeologous copies on 2AL and 2BL, respectively), *TaCRT2* on 5DL (with two additional homeologous copies on 4AL and 5BL, respectively) and *TaCRT3-1* on 3AL (with two additional homeologous copies on 3BL and 3DL, respectively). The sizes of the ORFs were 1248, 1287 and 1287 bp, the corresponding protein MWs were 47.2, 48.3 and 50.3 kDa and the protein isoelectric points were 4.32, 4.34 and 8.73, respectively. The proteins encoded by these three genes presented the typical domain patterns of CRT proteins [23]: a conserved N-terminal domain (N-domain), a proline-rich region (P-domain), a highly acidic region (C-domain) at the C-terminus and a C-terminal region ending with the ER-retention signal HDEL (Fig 1A). The P- and C-domains play a critical role in determining the Ca^2+^ storage capacity of the ER, and the N-domain might participate in the chaperone functions of the protein [37]. Using the PSORT function (http://psort.nibb.ac.jp/), it was found that a potential N-terminal ER import signal sequence (SS region) exists in all three proteins (Fig 1A) and that the C-terminal regions of these three proteins end with the ER-retention signal HDEL [23].

The amino acid sequences of the three wheat CRT proteins were then aligned with 23 plant CRT sequences and one *M*. *fuscata* CRT sequence. A phylogenetic tree was constructed based on the amino acid sequences and demonstrated the existence of two main clades (Fig 1B). *TaCRT1* and *TaCRT2* fell within one clade (group II in Fig 1B), and *TaCRT3* was grouped into another clade (group I in Fig 1B); this finding is indicative of evolutionary conservation among the members of different clades. In each clade, CRT proteins from monocot and dicot species were clearly separated as sub-groups, which clustered distinctively. Within the first clade, *TaCRT1/2* were grouped into the monocot sub-group together with *CRT1/2* from barley, *CRT1/2* from maize and *CRT1* from rice, and these proteins were clearly related to *CRT1/2* from barley. Interestingly, *TaCRT2* was most closely related to *CRT1* from rice; moreover, *TaCRT3-1* was paired with an orthologous *CRT3* from rice. Within the second clade, *TaCRT3-1* was grouped into the monocot sub-group together with *CRT3s* from maize and rice. Dedicated nucleotide and amino acid sequence alignments showed, respectively, 95% (nucleotide) and 96% (amino acid) identity between *TaCRT3-1* and a previously submitted wheat CRT (EF452301) [18], and 99% (nucleotide and amino acid) identity between *TaCRT3-1* and another previously submitted wheat CRT (HM037186) [19]. This means that our *TaCRT3-1* was identical to HM037186, and homeologous to EF452301, which has been showed to be involved in defense (yellow rust infection) responses and stress (dehydration) resistance, but *TaCRT1/2* have never been studied in wheat so far.

### Expression Patterns of *TaCRT* Genes

**Fig 2:**
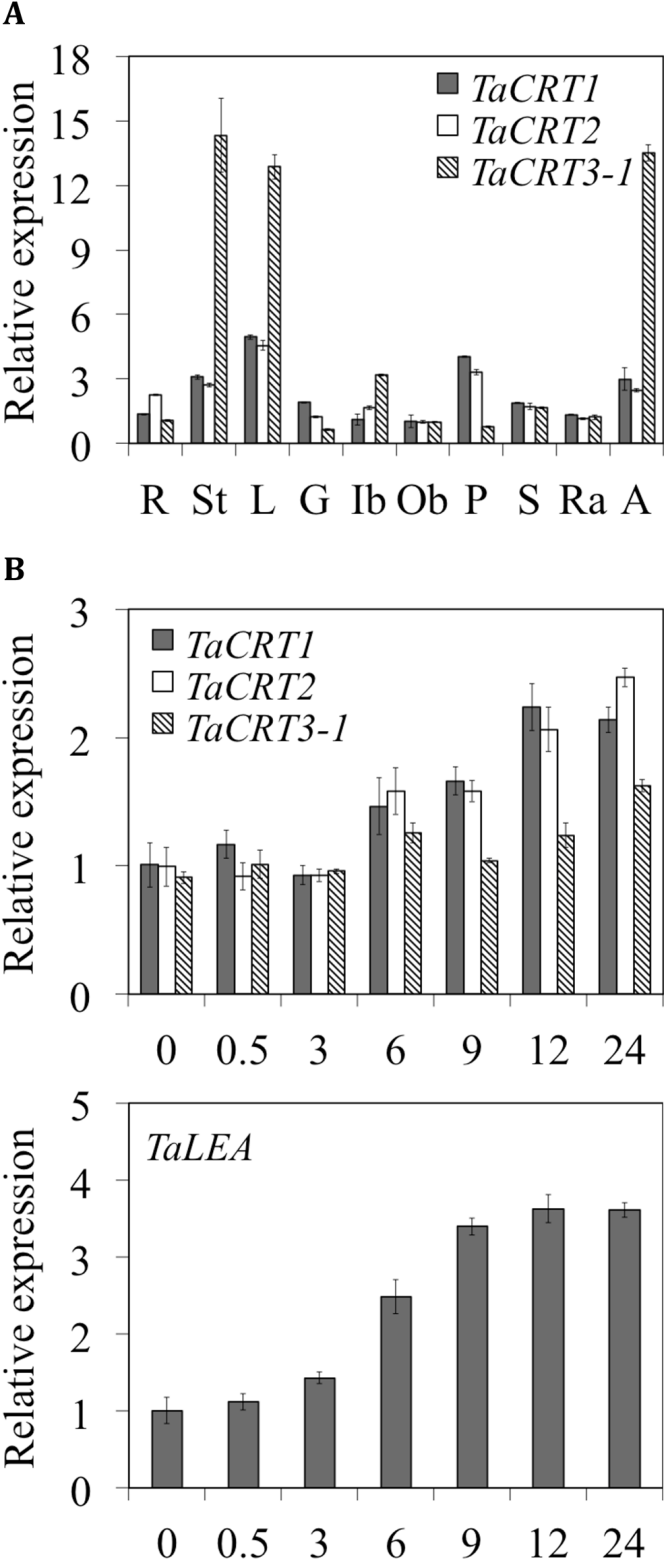
Expression of *TaCRT* genes and their response to salt stress in wheat. (A) Expression of *TaCRT* genes in various wheat tissues collected from field-grown plants at 15 days after anthesis, as measured using quantitative real-time PCR. R, seedling roots; L, leaves; St, spikes; G, glume; Ib and Ob, inner bract and outer bract, respectively; P and S, pistil and stamen, respectively; Ra and A, rachis and awn, respectively. (B) Expression patterns of *TaCRT* and *TaLEA* genes in wheat seedling roots after treatment with 250 mM. ‘0’ represents no treatment. Error bars represent the standard deviation of results obtained for three replicate experiments.

qRT-PCR was performed to determine the expression patterns of the *TaCRT* genes in different wheat tissues. The three *TaCRT* genes were expressed in all tested organs (Fig 2A); however, there was an obvious difference in transcript abundance. *TaCRT1* and *TaCRT2* transcripts were mainly detected in stems, leaves, pistils and awns at similar levels; *TaCRT3-1* showed ~4- to 5-fold increase in stems, leaves and awns, and ~1.5- to 5-fold decrease in roots, glumes and pistils compared to *TaCRT1/2*, suggesting that *TaCRT3-1* and *TaCRT1/2* are functionally distinct during wheat growth.

We examined the *TaCRT* expression patterns in response to treatment with 250 mM NaCl for various times. The transcript levels of the three *TaCRT* genes showed ~1.2- to 1.5-fold increase 6 h after the treatment, suggesting that these genes are up-regulated by NaCl stress (*TaCRT1* appeared to be induced or up-regulated 0.5 h after the NaCl treatment; Fig 2B). However, under the NaCl treatment, the expression pattern of *TaCRT3-1* was obviously different from those of *TaCRT1* and *TaCRT2* in roots. Likewise, the expression of *TaCRT1* and *TaCRT2* was more sensitive to NaCl stress and resulted in significantly higher transcript levels than those of *TaCRT3* 6–24 h after the NaCl stress.

The expression pattern of the wheat late embryogenesis abundant (LEA) protein gene *TaLEA* was analyzed in parallel on the same sets as a positive control (Fig 2C). LEA has previously been shown to be up-regulated by NaCl treatment in various plants [77–81].

### *TaCRT1* Encodes a Calcium-binding Protein

**Fig 3:**
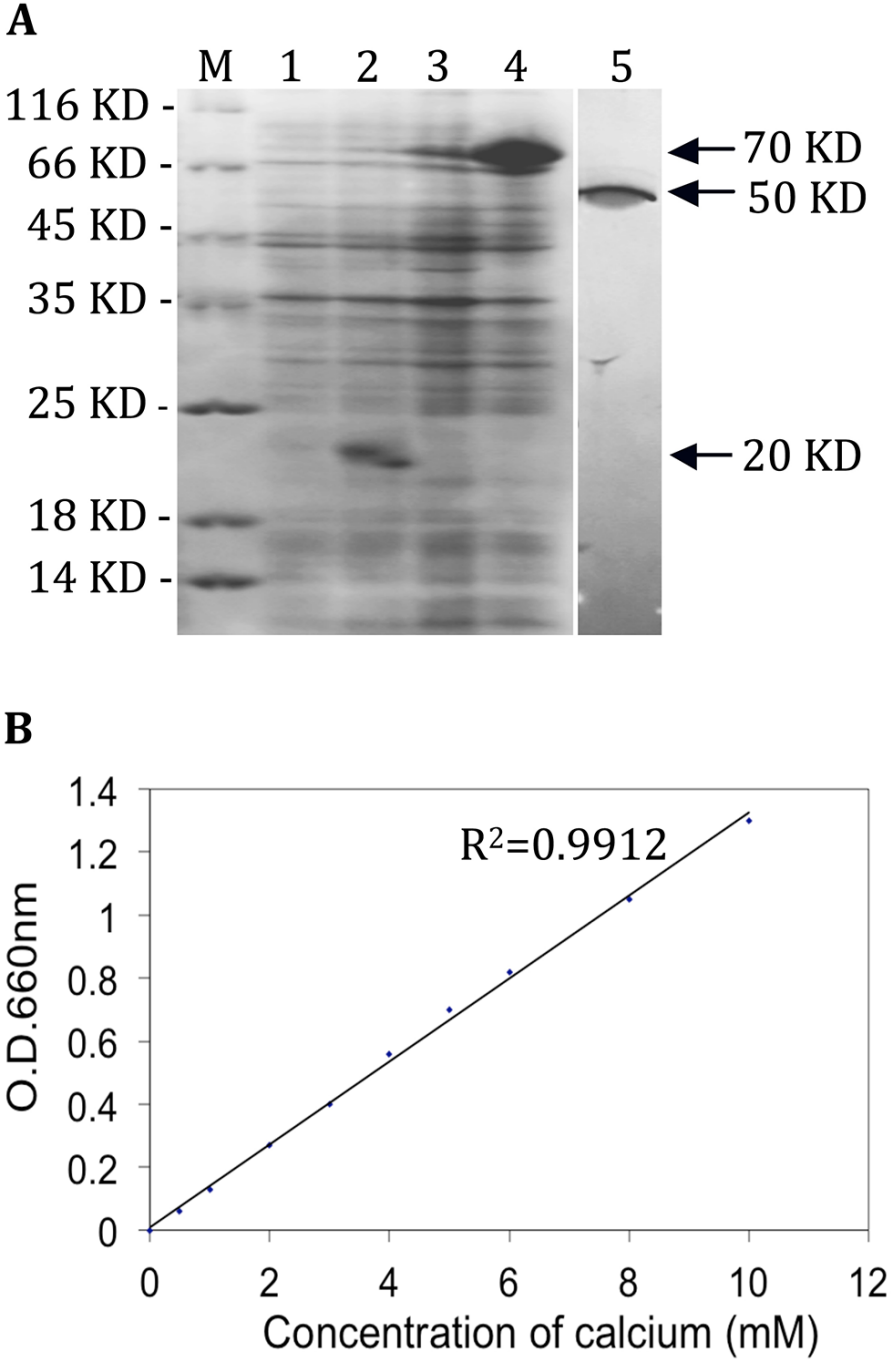
Purification of the 6xHis:TaCRT1 protein (A) and Scatchard plot of calcium binding to the affinity-purified TaCRT1 protein (B). The 6xHis and 6xHis:TaCRT1 proteins isolated from non-induced cells (lanes 1 and 3) and IPTG-induced cells (lanes 2 and 4) and purified TaCRT1 proteins obtained after thrombin cleavage (lane 5) were resolved on an SDS-PAGE gel and stained with Coomassie Blue. Lanes 1–4 contain 2 *μ*g of protein each.

The complete ORF of *TaCRT1* was cloned in a pET32a vector and expressed in *E*. *coli*. The recombinant protein was produced in *E*. *coli* after induction with IPTG. The 6xHis:TaCRT1 protein migrated on a SDS-PAGE gel with a mobility corresponding to a protein of approximately 70 kD (Fig 3A, lane 4), and the purified *TaCRT1* protein, after thrombin cleavage, migrated with a mobility corresponding to a protein of approximately 50 kD (Fig 3A, lane 5), a weight that is slightly larger than the predicted molecular mass (47.2 kD). This difference might be due to glycosylation of the calreticulin. For the 6xHis-only control, the expected band at a position corresponding to 20 kDa was observed (Fig 3A, lane 2). The calcium binding assay showed that the *TaCRT1* protein bound approximately 33.3 mol of Ca^2+^ per mol of protein (Fig 3B). Taken together, these results provide direct evidence that the gene product of *TaCRT1* is an authentic calcium-binding protein.

### *TaCRT1* Overexpression Improves Salt Tolerance and Enhances Antioxidant Enzyme Activities in Tobacco

**Fig 4:**
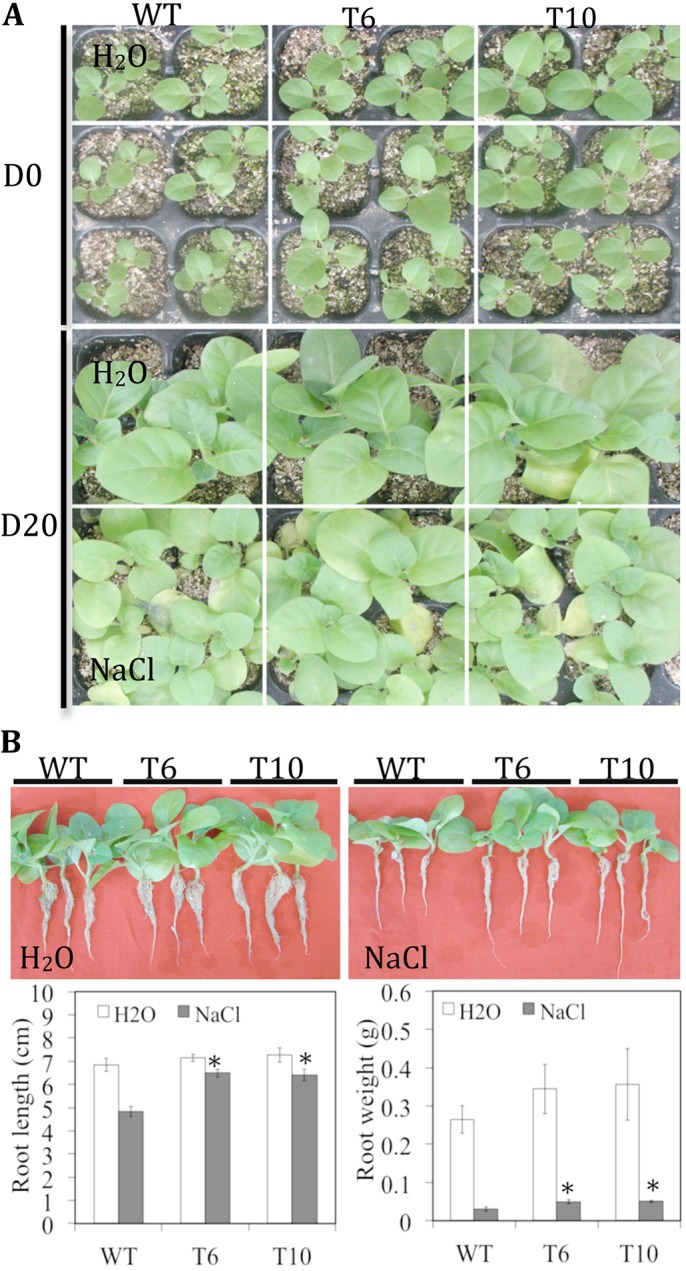
Overexpression of *TaCRT1* enhanced salt stress tolerance in transgenic tobacco plants. (**A**) Response of WT and transgenic tobacco lines to irrigation with 150 mM NaCl every 2 d. (**B**) Root length and fresh root weight of WT and transgenic tobacco lines treated with 150 mM NaCl. WT represents non-transgenic tobacco; T6 and T10 represent transgenic tobacco lines. Error bars represent the standard deviation of results obtained for three replicate experiments; asterisks indicate significant differences from WT plants at *P* = 0.05.

The growth of T_3_ transgenic tobacco (T6 and T10) and WT seedlings exposed to 150 mM NaCl stress was examined (Fig 4). The *TaCRT1* transgenic seedlings exhibited significantly enhanced shoot growth (or leaf area) and root length (or root fresh weight) compared with WT plants after 20 days of exposure to 150 mM NaCl. In contrast, only small differences were observed between the transgenic and WT seedlings on day 0 (no treatment) or on day 20 under normal growth conditions (irrigated with water; Fig 4A and 4B).

**Fig 5:**
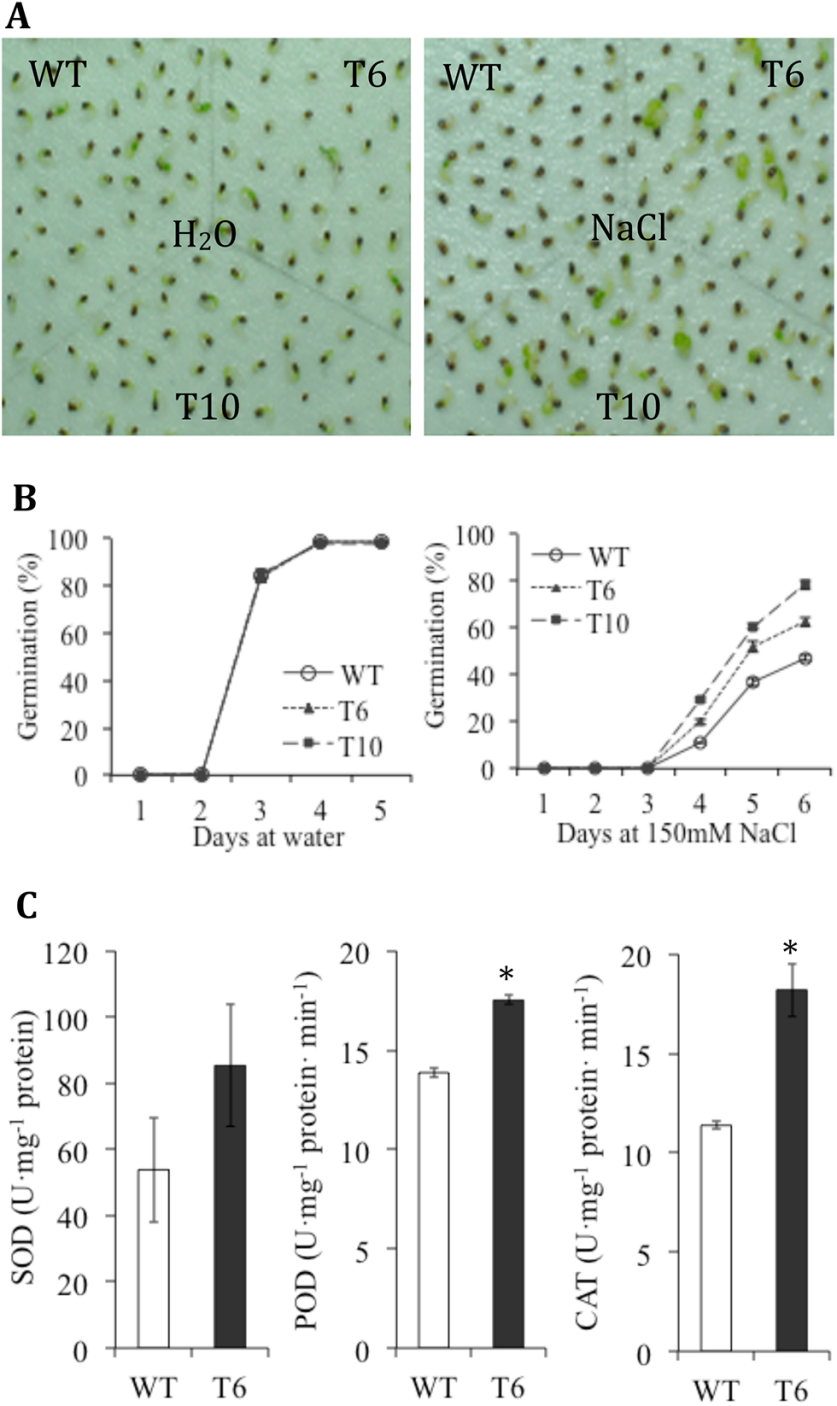
Germination rates of *TaCRT1-*overexpressing tobacco plants and WT plants under salt stress and activities of antioxidant enzymes in the roots of transgenic and non-transgenic tobacco. (**A**) Germination at 25°C ambient temperature of seeds sown on filter papers saturated with water or with 150 mM NaCl. Photographs were taken 6 d after sowing. (**B**) Germination rate from day 0 to day 6 after sowing. WT represents non-transgenic tobacco; T6 and T10 represent transgenic tobacco lines. Error bars represent the standard deviation of results obtained for three replicate experiments. (**C**) Activities of antioxidant enzymes SOD, POD and CAT in the roots from potted WT and transgenic (T6) tobacco under normal growth conditions.

In addition, the germination of T_3_ transgenic (T6 and T10) tobacco seeds and WT seeds were tested in the presence of NaCl. The *TaCRT1* transgenic seeds exhibited superior germination efficiency over WT seeds in the presence of 150 mM NaCl (Fig 5A). The mean germination rate from day 0 to day 6 after sowing showed that the germination of both WT and transgenic seeds was delayed at least one day or more by the treatment with 150 mM NaCl, compared with the germination of seeds in unsalted water (Fig 5B). Moreover, the *TaCRT1* transgenic seeds exhibited greater tolerance to 150 mM NaCl (beginning from day 3) than WT seeds, as demonstrated by their higher germination rate.

The activities of antioxidant enzymes such as SOD, POD and CAT were measured in the roots from potted WT and transgenic tobacco (T6) under normal growth conditions. The transgenic line showed higher and SOD, POD and CAT activities in roots than WT plants (Fig 5C), but the difference was statistically not significant for SOD activity. These results suggested that the overexpression of *TaCRT1* can enhance the antioxidant enzyme activities in tobacco.

### Overexpression of Partial *TaCRT1* Segments Increases the Salinity Tolerance in Transgenic Tobacco Plants

**Fig 6:**
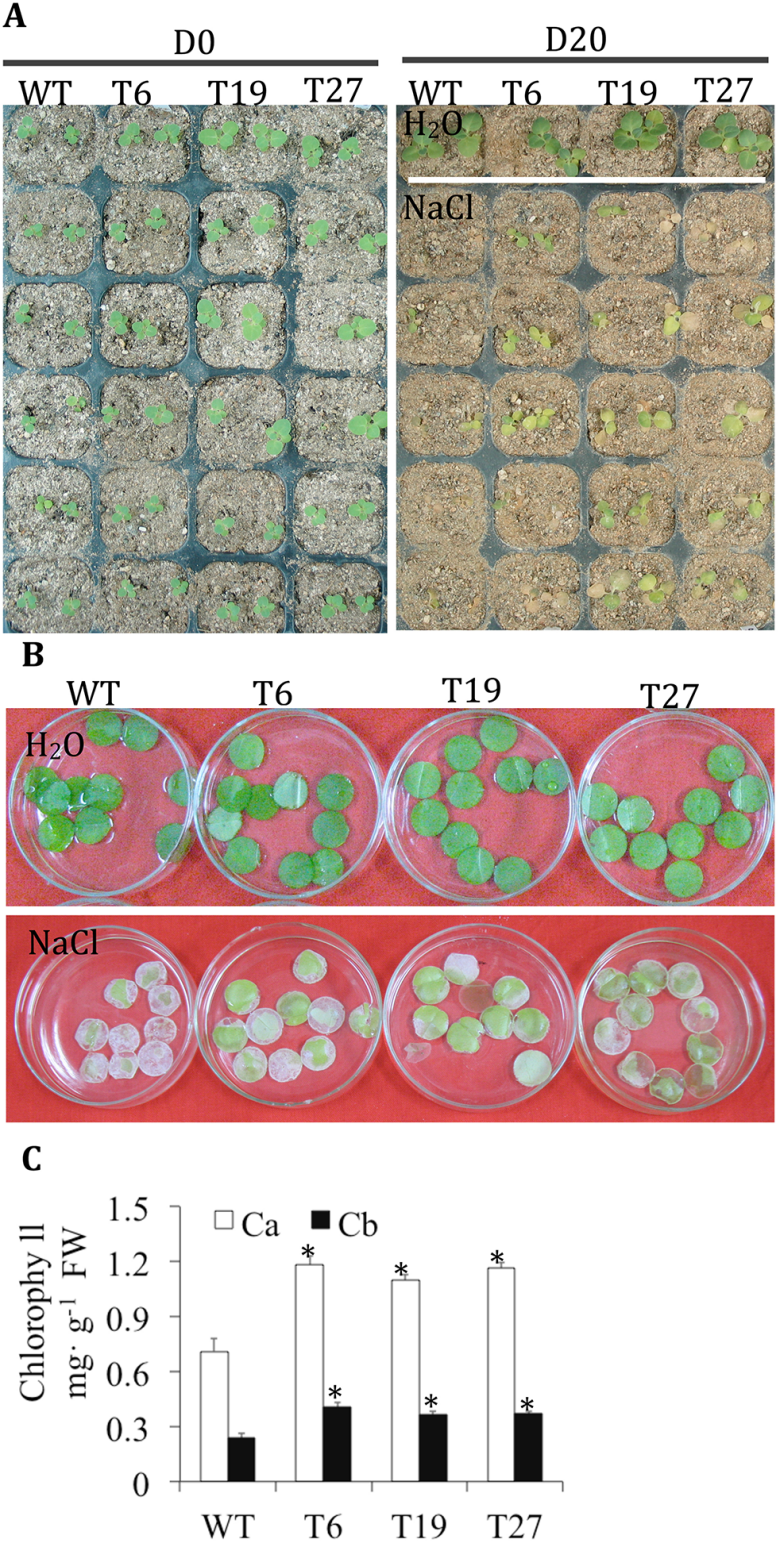
*TaCRT1* segments in transgenic tobacco exhibited greater chlorophyll content. (**A**) Phenotypes of transgenic tobacco lines under irrigation with 250 mM NaCl every 2 d. (**B**) Response of leaf discs obtained from WT and transgenic tobacco lines floating on a 400 mM NaCl solution. (C) Chlorophyll content of leaf discs obtained from WT and transgenic tobacco lines. WT represents non-transgenic tobacco; T6, T19 and T27 represent transgenic tobacco lines. D0 represents no treatment, D20 represents NaCl treatment for 20 d; Ca: chlorophyll a; Cb: chlorophyll b. Error bars represent the standard deviation of results obtained for three replicate experiments; asterisks indicate significant differences from WT plants at *P* = 0.05.

The growth of T_3_ transgenic tobacco seedlings of T6 (entire *TaCRT1*), T19 (omission of N-domain), T27 (omission of N- and P-domain) and WT seedlings was examined under exposure to 250 mM NaCl stress (Fig 6A). In each case, the growth of the transgenic seedlings was indistinguishable from that of WT seedlings grown under normal conditions. The results showed that the transgenic lines overexpressing the entire *TaCRT1* gene or partial *TaCRT1* segments exhibited better tolerance to salinity stress than their WT counterparts.

Leaf disc assays were also performed to determine the salinity tolerance of WT and T_3_ transgenic plants (T6, T19 and T27). Leaf discs from WT plants began to turn yellow after 3 days and were completely bleached after 5 days of treatment in a 400 mM NaCl solution; in contrast, leaf discs from T6, T19 and T27 plants exhibited greater tolerance under the same conditions (Fig 6B). The contents of chlorophyll a and b in the T6, T19 and T27 transgenic plants were significantly higher than the chlorophyll contents of WT plants (Fig 6C).

## Discussion

CRT is a widespread ER protein involved in many plant cellular processes, such as protein folding and calcium homeostasis [53, 55]. Dicot *CRT* genes have been well characterized, but monocot *CRT* genes remain poorly understood. In this study, three cDNAs from wheat, namely *TaCRT1*, *TaCRT2* and *TaCRT3-1*, which encode different CRT proteins, were identified (Fig 1A). The derived proteins contained domain patterns that are typical of plant CRTs [23]. Comparison of the putative wheat *CRT* sequences with *CRT*s from other plant species revealed that plant *CRT*s can be divided into two distinct groups: the *CRT1/2* group and the *CRT3* group (Fig 1B), as described in previous studies [56].

TaCRT1/TaCRT2 and TaCRT3-1 have orthologous isoforms in other species (Fig 1B). Sequence analysis showed that TaCRT1/TaCRT2 exhibits higher sequence identity with CRT1/CRT2 of barley and maize and with CRT1 of rice, whereas TaCRT3-1 exhibits high sequence identity with CRT3s of monocots such as rice and maize and with CRT3s of dicots such as *Arabidopsis*, rapeseed, grape and *M*. *truncatula* (Fig 1B). These findings suggest that at least three different *CRT* genes occur in the hexaploid wheat genome and that the isolated cDNAs of *TaCRT1*, *TaCRT2* and *TaCRT3-1* encode wheat CRT1, CRT2 and CRT3 isoforms, respectively. Because wheat is hexaploid, each of the three cloned wheat *CRT* genes has three homeologous copies in the wheat genome: *TaCRT1* on chromosome arm 2DL with two additional homeologous copies on 2AL and 2BL, respectively, *TaCRT2* on 5DL with two homeologous copies on 4AL and 5BL, respectively, and *TaCRT3-1* on 3AL with two homeologous copies on 3BL and 3DL, respectively. One of the three *TaCRT2* homeologous copies was located on a 4AL/5AL translocated chromosomal segment [82].

Similarly, our analysis suggests that the *TaCRT1*/*TaCRT2* and *TaCRT3-1* isoforms result from an early duplication event in wheat that might predate the evolutionary split of plants into dicots and monocots [83], possibly even the split between the animal and plant kingdoms [84]. In addition, Fig 2B shows that the *CRT1/2* genes evolved faster than the *CRT3* gene in plants.

Interestingly, CRTs were implicated in plant growth and development [8] due to their role in regulating calcium signaling and assisting protein folding [85]. In addition, the expression of *CRT* genes is up-regulated by a wide range of developmental and environmental stimuli, including cold [86, 87] and pathogens [88]. Jia et al. [18] found that the expression of a *CRT3* gene (EF452301) was significantly enhanced by PEG-induced drought stress in wheat seedlings. An et al. [19] found that the expression of another *CRT3* gene (HM037186) could be induced by *Puccinian striiformis* infection and cold treatment, but suppressed by dehydration in wheat seedlings. In this study, wheat *CRT1/CRT2* and *CRT3* differed in their expression patterns during plant development (Fig 2A) and were all up-regulated by NaCl salt stress (Fig 2B). Moreover, the *CRT1* and *CRT2* were induced at higher levels than *CRT3* under the same salt stress (Fig 2B). The up-regulation of *CRTs* is considered a conserved self-protection mechanism that was acquired during a long-term evolutionary process and that is likely to facilitate the survival of plants under unfavorable osmotic conditions [18].

In plants, the *Arabidopsis* AtCRT1a protein and several other CRT proteins have already been shown bind calcium [53,55]. The cDNA of *TaCRT1* was expressed in *E*. *coli*, and the purified protein was confirmed to possess Ca^2+^-binding properties (Fig 3A and Fig 3B), as expected for a typical CRT protein [22,23]. The result showing that *TaCRT1* possesses a Ca^2+^-binding function provides direct evidence that *TaCRT1* can sequester Ca^2+^. The highly conserved primary structure of TaCRT1/2 and TaCRT3 proteins among higher plants suggests that their fundamental functions might be conserved during the evolution of plants.

The overexpression of *TaCRT1* in tobacco enhanced seed germination (Fig 5) and significantly improved shoot growth (or leaf area) and root length (or root fresh weight) compared with WT plants under NaCl stress (Fig 4). In addition, similar phenotypes were observed in transgenic lines overexpressing partial *TaCRT1* segments that contained only the C-domain or the P- and C-domain (Fig 6A). Transgenic lines overexpressing the entire *TaCRT1* gene or partial *TaCRT1* gene segments exhibited better tolerance to the NaCl stress than their WT counterparts, revealing that the C-domains play essential roles in the response to NaCl stress, at least in plants. These results are consistent with those obtained in a previous study, which demonstrated that expression of the high-capacity calcium-binding domain of CRT increased the storage of bio-available calcium in plants [52]. Likewise, transgenic *Arabidopsis* plants expressing the C-domain of maize CRT exhibited significant resistance to drought, salt and heavy metal stresses [52]. To further consolidate our present results, the partial *TaCRT1* segments could be expressed in *E*. *coli* and the purified proteins might be also used for calcium-binding analysis to confirm their capacity of calcium-binding, respectively.

Salt stress can induce an increase in the production of cytotoxic reactive oxygen species (ROS) and oxidative damage in plants [89], and there is a constant need for efficient mechanisms to avoid oxidative damage to cells [90]. One of the most important plant strategies is to reduce the oxidative damage through improved antioxidant capacity. Antioxidant enzymes, such as SOD, POD and CAT etc. catalyze the scavenging of ROS and combat the oxidative damages induced by stresses [91]. A correlation between antioxidant capacity and salinity tolerance has been demonstrated in several plant species [92]. Our results showed that the transgenic tobacco overexpressing *TaCRT1* gene displayed higher SOD, POD and CAT activities in roots than non-transgenic tobacco (Fig 5C). These suggested that *TaCRT1* might confer the salt stress tolerance by enhancing the activities of antioxidant enzymes, which in turn protected transgenic tobacco against ROS-mediated damage under salt stress.

Calcium is an essential second messenger that mediates plant responses to developmental and environmental clues, increasing evidence supports that calcium levels are altered in plant cells in response to salt stress [93]. In plants, CRT plays important roles in a variety of cellular processes including regulating the Ca^2+^ homeostasis and protein folding [50]. The results here presented demonstrated that *TaCRT1* encodes a calcium-binding protein containing the three-domain structure typical of calreticulin protein (Fig 1A). This suggested that the activities of antioxidant enzymes might be enhanced by Ca^2+^ signaling mediated by TaCRT1 protein.

Jia et al. [18] showed that transgenic tobacco plants overexpressing a wheat *CRT3* gene (EF452301) exhibited enhanced drought resistance by their capacity to maintain higher water use efficiency, water retention ability, relative water content, and lower membrane damaging ratio under water deficit condition. Tsou et al. [94] showed that the overexpression of maize *CRT1* could improve tolerance to both salt and drought stresses in *Arabidopsis*, and meanwhile increase the total plant Ca^2+^ by ~25% and the expression level of calcineurin B-like protein-interacting protein kinases 6 (*CIPK6*), which is a member of the *CIPK* gene family. Deng et al. [95] showed that the overexpression of wheat *CIPK14* exhibited higher CAT activity, while decreased amounts of H_2_O_2_ and malondialdehyde, and less ion leakage under salt stresses, which then lead to salinity and cold tolerance in tobacco. Deng et al. [70] showed that the overexpression of wheat *CIPK29* can also result in an increased salt tolerance, accompanied by an increase of the expression level and activities of CAT and POD under salt stress in tobacco. The work presented here suggested a link between *TaCRT1* and antioxidant enzymes. The overexpression of *TaCRT1* could increase Ca^2+^ level, which would trigger the Ca^2+^ signaling downstream targets, like *CIPK* genes. *CIPK* genes might then regulate appropriate downstream responses such as the changes in the expression of protein kinases, transcription factors, and antioxidant enzymes, etc. Further studies are needed to examine if overexpression of *TaCRT1* could also improve tolerance to other stresses, such as drought and cold in tobacco or other plants.

Our study also showed that the leaf discs of transgenic tobacco plants tolerate salt better than those of non-transgenic plants (Fig 6B). Moreover, chlorophyll content was significantly higher in transgenic plants than in WT plants (Fig 6C). These results suggest that *TaCRT1* overexpression might increase the chlorophyll content in leaves. This study constitutes the first demonstration of an increase in chlorophyll content triggered by the overexpression of *CRT* genes. Our results are consistent with the study of Wyatt et al. [52], in which they found that overexpression of the C-domain of maize *CRT1* could delay the loss of chlorophyll in transgenic *Arabidopsis* plants on media lacking external Ca^2+^.

In the present study, we used the dicot tobacco (*N*. *tabacum*), one of the most-studied hosts for developmental and molecular genetic analysis, as host plant for *TaCRT1* overexpression analysis. Future studies with transgenic monocot wheats overexpressing *TaCRT1* or partial *TaCRT1* segments will help to consolidate the results of present work and elucidate better the *in vivo* mechanism of *TaCRT1-*mediated salinity tolerance in plants. As *TaCRT2* and *TaCRT1* have high sequence similarity to each other (Fig 1) and showed similar expression patterns clearly different from *TaCRT3-1* (Fig 2), further studies by overexpressing *TaCRT2* in tobacco or wheat will allow to know if the two genes had the same role in plant salinity tolerance.

In conclusion, three *CRT* genes, *TaCRT1*, *TaCRT2* and *TaCRT3-1*, were identified in hexaploid wheat. *TaCRT1* overexpression in tobacco improved salt tolerance, as manifested by a higher seed germination rate, significantly enhanced shoot growth (or leaf area) and root length (or root fresh weight) and greater chlorophyll content compared with WT plants. These results suggest that *CRT* genes are potential targets for improving environmental stress resistance in agricultural crops such as wheat and tobacco through genetic manipulation.

## Supporting Information

S1 Fig Detection and expression analysis of *TaCRT1* in transgenic tobacco plants. (A): PCR with genomic DNA of young leaves. (B): RT-PCR with root tissues. WT, non-transgenic tobacco; P, positive plasmid DNA; T6, T10, T19, T27, T_3_ transgenic tobacco plants.
(TIF)

S1 Table Primers used to isolate the *TaCRT* genes. (PDF)

S2 Table Primers used for quantitative real-time PCR. (PDF)

S3 Table Primers used to construct of *TaCRT* plant expression vector and transgenic plants detection. (PDF)

S4 Table Protein sequences used to construct of phylogenetic tree. (PDF)
