# Supplementary figures and images for: Overexpression of a Triticum aestivum Calreticulin gene (TaCRT1) Improves Salinity Tolerance in Tobacco
Source: PLoS One. 2015 Oct 15;10(10):e0140591. doi: 10.1371/journal.pone.0140591 (PMC4607401; doi:10.1371/journal.pone.0140591)

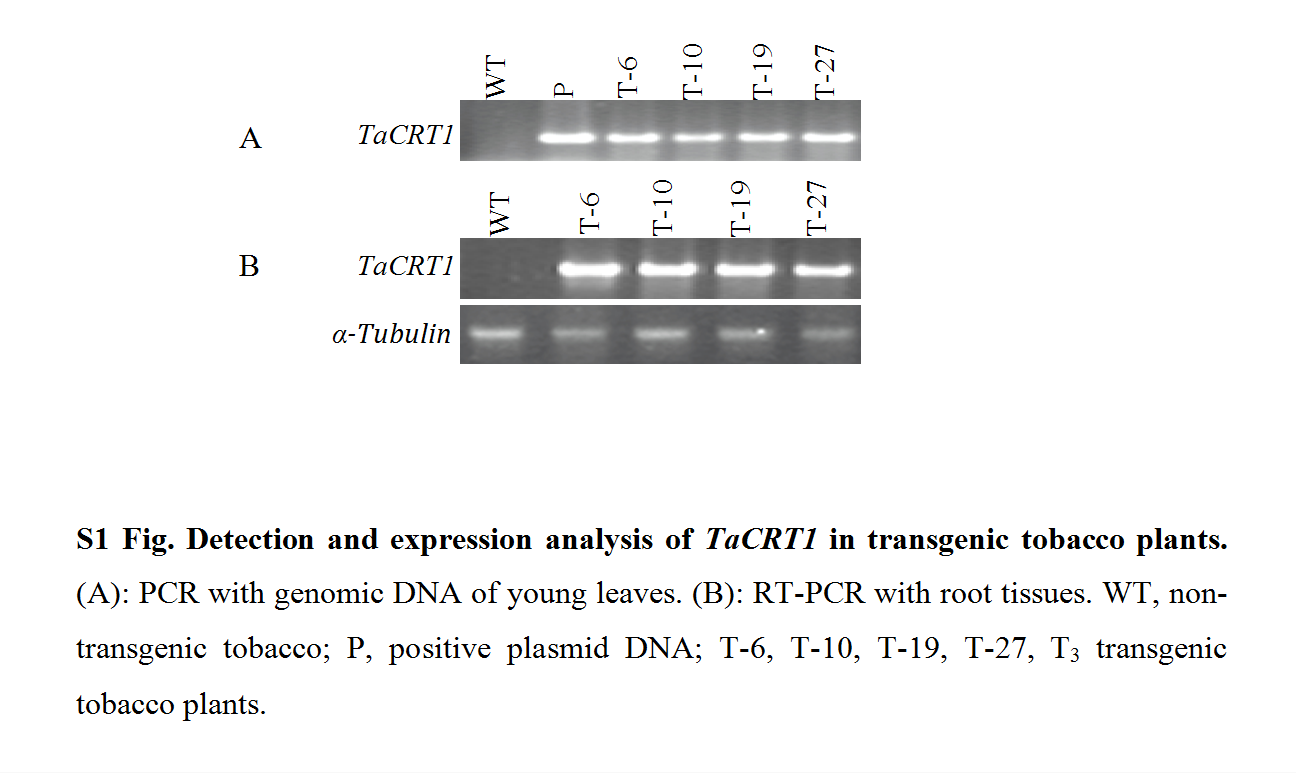

Supplement: S1 Fig — (A): PCR with genomic DNA of young leaves. (B): RT-PCR with root tissues. WT, non-transgenic tobacco; P, positive plasmid DNA; T6, T10, T19, T27, T3 transgenic tobacco plants. (TIF) [file pone.0140591.s001.tif]
