# Supplementary material for: Overexpression of a Triticum aestivum Calreticulin gene (TaCRT1) Improves Salinity Tolerance in Tobacco
Source: PLoS One. 2015 Oct 15;10(10):e0140591. doi: 10.1371/journal.pone.0140591 (PMC4607401; doi:10.1371/journal.pone.0140591)
Supplement: S1 Table — (PDF) [file pone.0140591.s002.pdf]

S1 Table. Primers used to isolate the *TaCRT* genes

Gene	Sequence of primer pairs (5′-3′)
TaCRT1	GAAGGCAGTAGAAATGGGTTCCG/ GACCCCACGGTGATGTTGTTCTTC
TaCRT2	GTTTTTGGTTGCCAGGGTTCG/ CAGAGAAAATGTTCTTCTAATTGCTGG
TaCRT3-1	AGCCCACCACCACTTCCTCGT/ GTGGAATTGT CTGTCCTGCTGTCAC
