# Supplementary material for: Overexpression of a Triticum aestivum Calreticulin gene (TaCRT1) Improves Salinity Tolerance in Tobacco
Source: PLoS One. 2015 Oct 15;10(10):e0140591. doi: 10.1371/journal.pone.0140591 (PMC4607401; doi:10.1371/journal.pone.0140591)
Supplement: S2 Table — (PDF) [file pone.0140591.s003.pdf]

S2 Table. Primers used for quantitative real-time PCR

Gene	Sequence of primer pairs (5′-3′)
TaCRT1	TCTGATGACGAGAAGCACGATGAGC/ GAGACAACTAATAAATCCTGGCAGCGG
TaCRT2	GGATGATGAGGAAGATGGTGAATGGAC/ CAGGCTGTCAAAAGCGTAGATGTAAGG
TaCRT3-1	TTACAAGGACAGATACAAAAGACGCAACAG/ TCCCTCACACGAGACAAGAAACACTTC
TaLEA	CAGGAGGAAGAAGGGGATGAAG/ ACACGGGGTCCACACATACG
α-Tubulin	ATCTCCAACTCCACCAGTGTCG/ TCATCGCCCTCATCACCGTC
