# Supplementary material for: Overexpression of a Triticum aestivum Calreticulin gene (TaCRT1) Improves Salinity Tolerance in Tobacco
Source: PLoS One. 2015 Oct 15;10(10):e0140591. doi: 10.1371/journal.pone.0140591 (PMC4607401; doi:10.1371/journal.pone.0140591)
Supplement: S3 Table — (PDF) [file pone.0140591.s004.pdf]

S3 Table. Primers used for construction of *TaCRT* plant expression vector and transgenic plants detection

TaCRT1 segments	Sequence of primer pairs (5′-3′)
**ORF**	CTTAAGATCTCGGGGTAAAGGCTTCCAC/TATTGGATCCGAGCTCATCGTG
**SS region**	CTGAACTAGTCGGGGTAAAGGCTTCCAC/ ACTATCTAGAGACGTCGGCGCTGAC
**P-, C-domain and HDEL**	CTACTCTAGAATGGATATTCTTCCTCCC/ TATTGGATCCGAGCTCATCGTG
**C-domain and HDEL**	CGCATCTAGAATGGATGATCCTTACATCTA/ TATTGGATCCGAGCTCATCGTG

The underlined sequences represented the inserted *Bgl*II, *Spe*I, *Xba*I and *BamH*I sites, respectively.
