# Supplementary material for: Overexpression of a Triticum aestivum Calreticulin gene (TaCRT1) Improves Salinity Tolerance in Tobacco
Source: PLoS One. 2015 Oct 15;10(10):e0140591. doi: 10.1371/journal.pone.0140591 (PMC4607401; doi:10.1371/journal.pone.0140591)
Supplement: S4 Table — (PDF) [file pone.0140591.s005.pdf]

S4 Table. Protein sequences used for construction of phylogenetic tree

> T. aestivum CRT1
-----MAIR-RGSSCAVLAL---LALASVAAVSADVFFQEK---FEDGWESRWVKSEWKK
DENM-AGEWNHTSGKWHGDAE-DKGIQTSEDYRFYAISAEYPEFSNKDKTLVLQFTVKHE
QKLDCGGGYVKLLGGDVDQKKFGGDTPYSIMFGPDICGYSTKKVHTILTKDGKNHLIKKD
VPCETDQLSHVYTLIIRPDATYSILIDNEEKQTGSIYEHWDILPPKEIKDPEAKKPEDWD
DKEYIPDPEDVKPEGYDDIPKEVTDPDAKKPEDWDDEEDGEWTAPTIPNPEYKGPWKQKK
IKNPNYQGKWKAPMIANPDFKDDPYIYAFDSLKYIGIELWQVKSGTLFDNILITDDAALA
KTFAEETWAKHKEAEKAAFDAAEK-KKEEEDASKASED-DDDLDDEEADDED-KDDKAGS
DAE-------------SDDEK-HDEL
> T. aestivum CRT2
-----MAVLARSAAAVAALA------LLASAVAGEVFFQEK---FDDGWEDRWVKSEWKK
EDNT-AGEWNHTSGKWNGDAD-DKGIQTSEDYRFYAISAQYPEFSNKDKTLVLQFSVKHE
QDLDCGGGYVKLLPADVDQKKFGGETPYSIMFGPDICGYATKKVHAILTKNGKNHLIKKE
VPCETDQLTHVYTLILRPDATYSILIDNVEKQSGSVYDDWDILPAKKKRDPNAKKPEDWE
DEEFLPDPEDKKPEGYDDIPKEITEPDATKPEDWDDEEDGEWTAPTIPNPEYKGPWIQKK
IKNPNFKGKWKAPLIDNPEFKDDPYIYAFDSLKHIGIELWQVKSGTLFDNILITDDPEYA
KKFAEETWGKHKDAEKAAFDEAEK-KRLEEESANAKTEDNDDAADEDEGKAAGASDEENK
DASGDEKVEEISKDASSSSDK-KDEL
> T. aestivum CRT3-1
-MGSSRRRGDR--H-LKLLHRLLALSSLLLLASGEVIFEER---FEDGWETRWVKSDWKK
SEGK-AGMFKHTAGKYSGDPD-DKGIQTTIDARHFAISAKIPEFSNKGRTLVVQYSIKFE
QEIECGGGYIKLTSGYVNQKKYSGDTPYSLMFGPDICGTQTKKLHLILSYQGQNYPIKKD
LQCETDRLTHVYTFILRPDASYSLLVDNRERESGSMYTDWDILPPRKIKDVGAKKPKDWD
DREYIEDPDAVKPEGYDSIPREIPDPKDKKPDTWDDDDDGIWKPRRIPNPAYKGQWKRKK
IKNPNYKGKWKIPWIDNPEFEDDPDLYVLKPLKYIGIEVWQVKAGSVFDNILICDDPEYA
KQVADETWGANKEAEKEAFKEAEKERKAREDKEAQQAREEGERRRRERGDRHRGRDHYKD
RYKRRNRDHWDD---------YHDEL
> T. aestivum CRT3
MMGSSRRRGDRQLQ-FQLLHRLLALSSLLLLASGEVIFEER---FEDGWETRWVKSDWKK
SEGK-AGMFKHTAGKYSGDPD-DKGIQTTIDARHFAISAKIPEFSNKGRTLVVQYSIKFE
QKIECGGGYVKLMSGYVNQKKYSGDTPYSLMFGPDICGTQTKKLHLILSYQGQNYPIKKD
LQCETDRLTHVYPFILRPDASYSLLVDNRERESGSMYTDWDILPPRKIKDAGAKKPRDWD
DPEYIEDPDAVKPEGYDSIPKEIPDPKDKKPDEWDDDDDGIWKPRRIPNPAYKGPWKRKK
IKNPNYKGKWKIPWIDNPEFEDDPDLYVLKPLKYIGIEVWQVKAGSVFDNILICDDPEYA
KQVADETWGANKEAEKEAFEEAEKERKAREDKEAQQAREEGERRRRERGDRHRGRDHYKD
RYKRRNRDHWDD---------YHDEL
> N. plumbaginifolia Crt
--MATQRRANPSSLHLITVFSL-----LVAVVSAEVFFEES---FNDGWESRWVKSEWKK
DENM-AGEWNHTSGKWNGDAN-DKGIQTSEDYRFYAISAEFPEFSNKGKNLVFQFSVKHE
QKLDCGGGYMKLLSGDVDQKKFGGDTPYSIMFGPDICGYSTKKVHAILTYNDTNHLIKKE
VPCETDQLTHVYTFILRPDATYSILIDNVEKQSGSLYSDWDLLPPKTIKDPSAKKPEDWD
EKEFIDDPEDKKPEGYDDIPEEITDPDAKKPEDWDDEEDGEWTAPTIPNPEYKGPWKPKK
IKNPNYKGKWKAPLIDNPDFKDDPDLYVFPKLKYVGVELWQVKSGTLFDNIVICDDPEYA
KAIAEETWGKQKDAEKAAFEEAEK-KREEEESKAAPADSDAE-EDDDADDDSDDAD--DK
SESKDDEAH-------------DEL
> A. thaliana Crt3
-MGLPQNKLSFFCF-----FFLVSVLTLAPLAFSEIFLEEH---FEGGWKSRWVLSDWKR
NEGK-AGTFKHTAGKWPGDPD-NKGIQTYNDAKHYAISAKIPEFSNKNRTLVVQYSVKIE
QDIECGGAYIKLLSGYVNQKQFGGDTPYSLMFGPDICGTQTKKLHVIVSYQGQNYPIKKD
LQCETDKLNHFYTFILRPDASYSVLVDNKEREFGSMYTDWDILPPRKIKVKNAKKPEDWD
DREYIDDPNDVKPEGFDSIPREIPDRKAKEPEDWDEEENGLWEPPKIPNFAYKGPWKAKR
IKNPNYKGKWKNPWIDNPEFEDDPDLYVLKSIKYAGIEVWQVKAGSIFDNILICDDPAYA
RSIVDDYFAQHRESEKELFAEAEKERKAREDEEARIAREEGERRRKERDHRY--GDRRRR
YKRPNPRDYMDD---------YHDEL
> M. truncatula Crt3
---MAENAS------TELKMFVLFCLLLIQVSLSEVIFEER---FEDGWRSRWVRSDWKS

SEGK-AGSFKHTAGKWAGDPD-DKGIQTSNDAKHFAISAKIPEFSNKNRTLVFQYSIKFE
QEIECGGGYMKLLSGFVNQKKFGGDTPYSVMFGPDLCGTDTKKLHVIVSYQGQNYPVKKD
LQCETDKLTHFYTFILRPDATYSVLVDNRERDSGSLYTDWDILPPRKIKDLKAKKPADWE
DREYIEDPNAVKPEGYDSIPAEIPDPKAKEPDSWDEDEDGIWKRPKIPNPAYKGPWKRKK
IKNPNYKGKWKTPWIDNPEFEDDPDLYVLKPIKYVGIEVWQVKGGSVFDNILICDDPEYA
KQVVDEVFAN-REIEKEAFEEAEKVRKAQEEEEAQRAREDGERRRKERGYDRH-RDRHRD
RYRKHRRDYMDD---------YHDEL
> B. napus Crt
-----MAKLT--LASSPWFLS-------VSWRSLPPLLSSRNALMMDGRTDGLNQSGRKR
TSLLGSGATLLEIGLVMLTIK-VSRPARTTDSTPSQLSSLN--SATRTKTLVCQFSVKHE
QKLDCGGGYMKLLSGDVDQKKFGGDTPYSIMFGPDICGYSTKKVHAILTYNDANHLIKKD
VPCETDQLTHVYTFILRPDATYTILVDNVEKQTGSLYSDWDLLPPKKIKDPSAKKPEDWD
EQEYIPDPEDKKPDGYDDIPKEIPDTDAKKPEDWDEEEDGEWTAPTIPNPEYMGEWKPKQ
IKNPNYKGKWEAPEIDNPDFKDDPELYVFPKLKYVGIELWQVKSGSLFDNVLICDDPDYA
KKLAEETWGKLKDAEKAAFDEIEK-KKEEEESKDAPAETDGEDEAEEDEG--EESDTESK
T-EAKSEVSEET---AEKDATAHDEL
> A. thaliana Crt2
-----MAKMIPSLVSLILIGL-------VAIASAAVIFEER---FDDGWENRWVKSEWKK
DDNT-AGEWKHTAGNWSGDAN-DKGIQTSEDYRFYAISAEFPEFSNKDKTLVFQFSVKHE
QKLDCGGGYMKLLSGDVDQKKFGGDTPYSIMFGPDICGYSTKKVHAILTYNGANHLIKKD
VPCETDQLTHVYTFILRPDATYSILIDNVEKQTGSLYSDWDLLPPKKIKDPSAKKPEDWD
EQEYISDPEDKKPDGYDDIPKEIPDTDSKKPEDWDDEEDGEWTAPTIPNPEYMGEWKPKQ
IKNPNYKGKWEAPLIDNPDFKDDPELYVFPKLKYVGLELWQVKSGSLFDNVLICDDPDYA
KKLADETWGKLKDAEKAAFDEAEK-KNEEEESKDAPAESDAEDEPEDDEG-GDDSDSESK
AEETKSVDSEET---SEKDATAHDEL
> B. vulgaris Crt
---MENRGRNPSFLSLLLLLS------LFAIASAKVFFEER---FEDGWEKRWVKSEWKK
DESM-AGEWNYTSGKWNGDAN-DKGIQTSEDYRFYAISAEFPEFSNKDNTLVFQFSVKHE
QKLDCGGGYMKLLSGEVDQKKFGGDTPYSIMFGPDICGYSTKKVHAIFNYNDTNHLIKKD
VPCETDQLTHVYTFILRPDATYSILIDNQEKQTGSLYTDWDLLPAKKIKDPEAKKPEDWD
DKEFIPDPEDKKPEGYDDIPAEITDPEAKKPEDWDDEEDGEWTAPTIPNPEYKGPWKAKK
IKNPNYKGKWKAPMIDNPEFKDDPELYVYPKLRYVGVELWQVKSGTLFDNVLVCDDPEYA
KQLAEETWGKQKDAEKAAFEELEK-KREEEETKDDPVESDAE-DEDEAEADDSDKDDADK
SDDKDDDQH-------------DEL
> B. rapa Crt3
-MRLTQNKLKPFH------LFLFSLLTLTPLAFSEIFFEEH---FEGGWKSRWVLSDWKR
NEGK-AGTFKHTAGKWPGDPD-NKGIQTYNDAKHYAISAKIQEFSNKNRTLVVQYSVKIE
QDIECGGAYIKLLSGYVNQKQFGGDTPYSLMFGPDICGTQTKKLHVILSYQGQNYPIKKD
LQCETDKLNHFYTFILRPDASYSVLVDNKEREFGSMYTDWDILPPRKIKVKNAKKPVDWD
DREYIDDPDDVKPEGYDSIPREIRDQKAEEPEDWDEEENGPWEAPKIPNPAYKGPWKAKK
IKNPNYKGKWKNPWIDNPEFEDDPDLYVLKPIKYIGIEVWQVKAGSIFDNILITDDPQYA
RAMVDDYFEQHRESEKELFAEAEKERKAREEE----------------------------

> G. max Crt1
---MAFRVRSPDLRSLFLLS-------LLSIASANVFFEER---FDDGWENRWVKSDWKK
DENV-AGEWNHTSGQWNGDAN-DKGIQTSEDYRFYAISAEYPEFSNKGKTLVFQFSVKHE
QKLDCGGGYMKLLSGDVDQKKFGGDTPYSIMFGPDICGYSTKKVHAILTYNNTNHLIKKD
VPCETDQLTHVYTFILRPDATYSILIDNVEKQTGSLYSDWDLLPPKTIKDPEAKKPEDWD
DKEYIPDPEDKKPEGYDDIPKELPDPEAKKPEDWDDEEDGEWTPPTIANPEYKGPWKAEK
IKNPNYKGKWKAPLIDNPDFKDDPDLYVFPNLKYVGIELWQVKSGTLFDNVLITDDPEYA
KQLVEETWGKHKDAEKAAFEEAEK-KREEEESKDDPVDSDADEEEEDADEAGNDSDAESK
TEAGEDTKE----------EGVHDEL
> N. tabacum Crt
----------------------------------EVFFEES---FNDGWESRWVKSEWKK
DENM-AGEWNHTSGKWNGDAN-DKGIQTSEDYRFFAISAEFPEFSNKGKNLVFQFSVKHE
QKLDCGGGYMKLLSGDVDQKKFGGDTPYGIMFGPDICGYSTKKVHAILTYNDTNHLIKKE
VPCETDQLTHVYTFILRPDATYSILIDNVEKQSGSLYSDWDLLPPKTIKDPSAKKPEDWD
EKEFIDDPEDKKPEGYDDIPEEITDPDAKKPEDWDDQEDGEWTAPTIPNPEYKGPWKPKK

IKNPNYKGKWKAPLIDNPDFKDDPDLYVFPNLKYVGVELWQVKSGTLFDNIVICDDPEYA
KAIAEETWGKQKDAEKAAFEEAEK-KREEEESKAAPADSDAE-EDDDADDDADDAD--DK
LESKDDEAH-------------DEL
> A. thaliana Crt1
-----MAKLNPKFISLILFAL-------VVIVSAEVIFEEK---FEDGWEKRWVKSDWKK
DDNT-AGEWKHTAGNWSGDAN-DKGIQTSEDYRFYAISAEFPEFSNKDKTLVFQFSVKHE
QKLDCGGGYMKLLSDDVDQTKFGGDTPYSIMFGPDICGYSTKKVHAILTYNGTNHLIKKE
VPCETDQLTHVYTFVLRPDATYSILIDNVEKQTGSLYSDWDLLPAKKIKDPSAKKPEDWD
DKEYIPDPEDTKPAGYDDIPKEIPDTDAKKPEDWDDEEDGEWTAPTIPNPEYNGEWKPKK
IKNPAYKGKWKAPMIDNPEFKDDPELYVFPKLKYVGVELWQVKSGSLFDNVLVSDDPEYA
KKLAEETWGKHKDAEKAAFDEAEK-KREEEESKDAPAESDAEEEAEDDDNEGDDSDNESK
SEETKEAEETKE---AEETDAAHDEL
> B. stolonifera Crt
-----MAIAERRSRSHLALR-------VRDRVSAEVFFEER---FEDGWESKWVKSDWKR
DENM-AGEWNFTSGKWNGDAN-DKGIQTSEDYRFYAISAAFPEFSNKGKTLVFQFSVKHE
QKLDCGGGYMKLLSGDVDQKKFGGDTPYSIMFGPDICGYSTKKVHAILTKGETNHLIKKD
VPCETDQLTHVYTFILRPDASYSILIDNVEKQSGSVYTDWDILPPKQIKDPEAKKPEDWE
DKEYIPDPEDKKPEGYDDIPKEITDPEAKKPEDWDDEEDGEWTAPTIPNPDYKGEWKPKK
IKNPNFKGKWKAPMIDNPDFKDDPDIYVFPKLKYVGIELWQVKSGTMFDNVLICDDPDYA
KKLAEETWGKNKDAEKAAFDEAEK-KKEEEEAKDDPTESDDEKPDEEGESDGEGDDESKD
IDNEEDDED-----------VHDEL
> H. vulgare Crt2
----------LLRRLAL------LALASVAAVAADVFFQEK---FEDGWESRWVKSEWKK
DENM-AGEWNHTSGKWHGDAE-DKGIQTSEDYRFYAISAEYPEFSNKDKTLVLQFTVKHE
QKLDCGGGYVKLLGGDVDQKKFGGDTPYGIMFGPDICGYSTKKVHTILTKNGKNHLIKKD
VPCETDQLSHVYTLIIRPDATYSILIDNEEKQTGSIYEHWDILPPKEIKDPEAKKPEDWD
DKEYIPDPEDVKPEGYDDIPKEVTDPDAKKPEDWDDEEDGEWTAPTIPNPEYKGPWKQKK
IKNPNYQGKWKAPMIANPDFQDDPYIYAFDSLKYIGIELWQVKSGTLFDNILITDDAALA
KTFAEETWAKHKDAEKAAFDEAEK-KKEEEDASKAGED-DDDLDDEDADDED-KDDKAGS
DAEDDK---------DSDDEK-HDEL
> H. vulgare Crt1
----------RIGKKALESR---LVCASVAAVAADVFFQEK---FEDGWESRWVKSEWKK
DENM-AGEWNHTSGKWHGDAE-DKGIQTSEDYRFYAISAEYPEFSNKDKTLVLQFTVKHE
QKLDCGGGYVKLLGGDVDQKKFGGDTPYGIMFGPDICGYSTKKVHTILTKNGKNHLIKKD
VPCETDQLSHVYTLIIRPDATYSILIDNEEKQTGSIYEHWDILPPKEIKDPEAKKPEDWD
DKEYIPDPEDVKPEGYDDIPKEVTDPDAKKPEDWDDEEDGEWTAPTIPNPEYKGPWKQKK
IKNPNYQGKWKAPMIANPDFQDDPYIYAFDSLKYIGIELWQVKSGTLFDNILITDDAALA
KTFAEETWAKHKDAEKAAFDEAEK-KKEEEDASKAGED-DDDLDDEDADDED-KDDKAGS
DAEDDK---------DSDDEK-HDEL
> M. truncatula Crt1
---MAIRVRNPNLLSLVLFS-------LLSIASAKVFFEER---FQDGWESRWVKSEWKK
DENL-AGEWNYTSGQWNGDAN-DKGIQTSEDYRFYAISAEFPEFSNKDNTLVFQFSVKHE
QKLDCGGGYMKLLSGDVDQKNFGGDTPYSIMFGPDICGYSTKKVHAILTYNDTNHLIKKD
VPCETDQLTHVYTFIIRPDATYSILIDNVEKQTGSLYSDWSLLPPKKIKDPEAKKPEDWD
DKEFIPDPEDKKPEGYDDIPKEVADPDAKKPEDWDDEEDGEWTAPTIANPEYKGPWKPKK
IKNPNYSGKWKAPLIDNPDFKDDPDIYVFPKLKYVGIELWQVKSGTLFDNVVITDDPEYA
KQVAEETWGKQKDAEKAAFEEAEK-KKEEEETKDDPVDSDAEEDEEDANEVSHDSDDESK
AEAGEDSDET-------NKDDVHDEL
> O. sativa Crt1/2
-----MAIRARSSSYAAAAVALALALASVAAVAGEVFFQEK---FEDGWESRWVKSEWKK
DENM-AGEWNHTSGKWNGDPE-DKGIQTSEDYRFYAISAEYPEFSNKDKTLVLQFSVKHE
QKLDCGGGYVKLLGGDVDQKKFGGDTPYSIMFGPDICGYSTKKVHTIFTKNDKNHLIKKD
VPCETDQLSHVYTLIIHPDATYTILIDNVEKQSGSIYEHWDILPPKQIKDPEAKKPEDWD
DKEYIPDPEDKKPEGYDDIPKEIPDPDAKKPEDWDDEEDGEWTAPTIPNPEYKGPWKQKK
IKNPNYQGKWKAPMIDNPDFKDDPYIYAFDSLKYIGIELWQVKSGTLFDNFLITDDPELA
KTFAEETWGKHKDAEKAAFDEAEK-KKEEEEAAKAGED-DDDLDDEDAEDEDKADEKADS
DAEDGK---------DSDDEK-HDEL
> O. sativa Crt3

-MG--SRSGGR----HRLFLRFIALSSLLLIAAGEVIFEER---FEDGWESRWVKSDWKR
SEGK-AGTFKHTAGRYSGDPD-DKGIQTTLDARHFAISAKIPEFSNKGRTLVLQYSIKFE
QDIECGGGYIKLMSGYVNQKKFSGDTPYSLMFGPDICGTQTKKLHLILSYQGQNYPIKKD
LQCETDKLTHVYTFILRPDASYSILVDNRERESGSMYTDWDILPPRKIKDVHAKKPKDWD
DREYIEDPDAVKPEGYDSIPKEIPDPKDKKPDTWDDDDDGIWKPRMIPNPAYKGPWKRKK
IKNPNYKGKWKIPWIDNPEFEDDPDLYVLKPLKYIGIEVWQVKAGSVFDNILICDDPEYA
RKAAEETWGANREAEKEAFEEAEKERKAREDKEAERAREEGERRRRERGDRHRGRD-YKD
RYKRRHRDHWDDD--------YHDEL
> P. armeniaca Crt
---MAFRVPNSSLLSLILLS-------LLAIASAKVFFEER---FEDGWDKRWVTSEWKK
DENL-AGEWNYTSGKWNGDPN-DKGIQTSEDYRFYAISAEFPEFSNKDKTLVFQFSVKHE
QKLDCGGGYIKLLSGDVDQKKFGGDTPYSIMFGPDICGYSTKKVHAILNYNNTNNLIKKD
VPCETDQLTHVYTFIIRPDATYSILIDNLEKQTGSLYSDWDLLPAKKIKDPEAKKPEDWE
DQEYIPDPEDKKPEGYDDIPKEITDPDAKKPEDWDDEEDGEWTAPTIPNPEYKGEWKPKK
IKNPNFKGKWKAPLIDNPEFKDDPELYVYPNLKYVGIELWQVKSGTLFDNILITDEPEYA
KQLAEETWGKQKDAEKAAFEELEK-KLQEEESKEDPVDSDAEDDDNEAEDGE-ESDSESK
PDSTEESAET--------EAEKHDEL
> P. taeda Crt
-----MAGRRSLLYAVFLLL-------FVTLVSAEVFFEER---FDDSWESRWVQSDWKK
DESL-AGDWVHTSGKWNGDPN-DKGIQTHTDYRFFAISAAYPEFSNKDKTLVLQFSVKHE
QKLDCGGGYVKLLSGEIDQKNFSGETPYSIMFGPDICGYSTKKVHTILSYKG-KHPIKKD
VPCETDQLTHVYTFILRPDATYSILIDNTDKQSGSLYKDWDLLPPKTIKDPNAKKPEDWD
DKEYIPDPEDKKPEGYDDIPKEIPDPDATKPEDWNDEEDGEWTAPTIANPEYKGPWKPKK
IKNPNYKGKWKAPMIDNPDFKDDPELYVFPNLKYLGIELWQVKSGTLFDNILISDDPEYA
KKLAEETWAKHKDAEKEAFDEAEK-KKEEEEKEKESDDEEDTDEKEEKSDDEDADELDDE
HKEADKKEHELDSEHKEEDKKEHDEL
> R. communis Crt
-------MANPKSLSLFLLS-------LLAIASAEVFFEER---FEDGWENRWVKSDWKK
DENT-AGEWNYTSGKWNGDPN-DKGIQTSEDYRFYAISAEFPEFSNKDKTLVFQFSVKHE
QKLDCGGGYMKLLSSSTDQKKFGGDTPYSIMFGPDICGYSTKKVHAILNYNDTNHLIKKE
VPCETDQLTHVYTLVIRPDATYSILIDNVEKQTGSLYTDWDLLPPKKIKDPEAKKPEDWD
EKEYIPDPEDKKPEGYDDIPKEIPDPDAKKPEDWDDEEDGEWTAPTIANPEYKGPWKPKK
IKNPNYKGKWKAPMIDNPDFKDDPEIYVYPNLKYVGIELWQVKSGTLFDNVLICNDPEYA
KQLAEETWGKNKDAEKAAFEEAEK-KKEEEESKDDPADSDADEDDDDADDTEGEDDGESK
SDAAEDSAE-----------DVHDEL
> V. vinifera Crt3
-MGFLSNSSGFSDMLKKLLLLPLLLSFLLTFSLSEVIFEER---FEDGWQSRWVKSDWKK
SEGK-AGSFKHTAGKWAGDPD-DKGIQTSTDARHFAISAKIPEFSNKNRTLVLQYSIRFE
QEIECGGGYIKLLSGFVNQKKFGGDTPYSVMFGPDLCGTQTKKLHVIVSYQGQNYPIKKD
LQCETDKLTHFYTFILRPDASYSVLIDNRERESGSMYSDWDILPPRKIKDTKAKKPADWD
DREYIEDPNDVKPEGYDSIPAEIPDPKAKEPDNWDEEEDGLWKPPKIPNPAFKGPWRRKK
IKNPNYKGKWKNQWIDNPEFEDDPDLYVLKPIKYVGIEVWQVKAGAIYDNILICDDPEYA
KQVVEEVFAH-RESEKEAFEEAEKVRKAREEEEAQRAREEGERRRRERGHDR--RYRDRE
RYRDRYRRVCIS---------YA---
> Z. mays Crt1
-----MAIR-KGSSYAVAAL---LALASVAAVAGEVFFQEK---FEDGWESRWVKSEWKK
DENM-AGEWNHTSGKWNGDAE-DKGIQTSEDYRFYAISAEYPEFSNKDKTLVLQFSVKHE
QKLDCGGGYVKLLGGDVDQKKFGGDTSYSIMFGPDICGYSTKKVHTILTKDGKNHLIKKD
VPCETDQLTHVYTLIIRPDATYSILIDNEEKQTGSIYEHWDILPPKKIKDPEAKKPEDWD
DKEYIPDPEDKKPEGYDDIPKEIPDPDAKKPEDWDDEEDGEWTAPTIPNPEYKGPWKQKK
IKNPNYQGKWKAPMIDNPDFKDDPYIYAFDSLKYIGIELWQVKSGTLFDNIIITDDPALA
KTFAEETWGKHKEAEKAAFDEAEK-KKEEEDAAKGGDDEDDDLEDEEDDEKA-DEDKADS
DAEDSK---------DSDDEKQHDEL
> Z. mays Crt2
-----MAIR-KGSSYAVAAL---LALASVAAVAGEVFFQEK---FEDGWESRWVKSEWKK
DENM-AGEWNHTSGKWNGDAE-DKGIQTSEDYRFYAISAEYPEFSNKDKTLVLQFSVKHE
QKLDCGGGYVKLLGGDVDQKTLGGDTSYSIISRPDISRYSTKKVHTILTKDGKNHLIKKD
VPCQTDQLTHVYTFIIRPDATYSILIDNEEKHTGSIYEHWDILPPKKIKDPEAKKPEDWD

```
DKEYIPDPEDKKPEGYDDIPKEIPDPDAKKPEDWDDEEDGEWTAPTIPNPEYKGPWKQKK
IKNPNYQGKWKAPMIDNPDFKDDPYIYAFDSLKYIGIELWQVKSGTLFDNIIITDDPALA
KTFAEETWGKHKEAEKAAFDEAEK-KKEEEDAAKGGDDEDDDLEDEEDDEKA-DEDKADS
DAEDGK---------DSDDEK-HDEL
> Z. mays Crt3
-MGTGRRGGGG----GGVFHRLLALSSLLLLASGEVVFEER---FEDGWETRWVESDWKR
SEGK-AGRFKHTAGRYSADPD-DKGIQTTIDARHFAISAKFPEFSNKNRTLVVQYSIKFE
QDIECGGGYIKLMSGYVNQKKFSGDTPYSLMFGPDICGTQTKKLHLILSYQGQNYPIKKD
LECETDKLTHVYTFILRPDASYSLLVDNRERESGSMYTDWDILPPRKIKDVHAKRPKDWD
DREYIEDPDEVKPEGYDSIPKQIPDPKDKKPDTWDEDEDGIWKPRMVSNPAYKGPWKRKR
IKNPNYKGKWKTPWIDNPEFEDDPDLYVLKPLKYIGIEVWQVKAGSVFDNILICDDPEYA
RKVVEETWGANREAEKEAFEEAEKERKAREDREGQKAKDDGGRHRIHR------------
RHKKHYRDHWDD---------YHDEL
> M. fuscata Crt
-----MLLSVPLLLGLLG----------LAAAEPAVYFKEQ-FLDGDGWTSRWIESKHKS
DFGK----FVLSSGKFYGDEEKDKGLQTSQDARFYALSASFEPFSNKGQTLVVQFTVKHE
QNIDCGGGYVKLFPNSLDQTDMHGDSEYNIMFGPDICGPGTKKVHVIFNYKGKNVLINKD
IRCKDDEFTHLYTLIVRPDNTYEVKIDNSQVESGSLEDDWDFLPPKKIKDPDASKPEDWD
ERAKIDDPTDSKPEDWD-KPEHIPDPDAKKPEDWDEEMDGEWEPPVIQNPEYKGEWKPRQ
IDNPDYKGTWIHPEIDNPEYSPDPSIYAYDNFGVLGLDLWQVKSGTIFDNFLITNDEAYA
EEFGNETWGVTKVSPGPDDEEQRL--KEEEEDKKRKEEEEAEDKEDDEDKDEDEEDEEDK
EEDEEEDVPG----------QAKDEL
```
